# Navigating Treatment Refusal: Behaviour Guidance for Down Syndrome Oral Health Management

**DOI:** 10.1155/2024/2966972

**Published:** 2024-06-18

**Authors:** Lila Sari, Risti Saptarini Primarti, Arlette Suzy Setiawan

**Affiliations:** ^1^Pediatric Dentistry Residency Program, Faculty of Dentistry, Universitas Padjadjaran, Bandung, Indonesia; ^2^Department of Pediatric Dentistry, Faculty of Dentistry, Universitas Padjadjaran, Bandung, Indonesia

## Abstract

**Background:**

Managing dental care in children with special health care needs poses distinct challenges. This case report explores these challenges within the context of a 9-year-old boy with Down syndrome (DS) facing dental treatment refusal.

**Case Report:**

Over ten months and 13 visits, a tailored approach was devised for the patient who presented with multiple cavities and retained primary teeth. Key strategies included gradually introducing dental procedures, including tooth brushing, intraoral examination, tooth preparation, extraction, and myofunctional therapy. Behaviour guidance techniques include tell-show-do, desensitization, positive reinforcement, and close collaboration between dental professionals and the patient's mother.

**Discussion:**

This methodical approach helped overcome the child's initial refusal without sedation or general anaesthesia, facilitating successful dental care.

**Conclusion:**

This case emphasizes the effectiveness of patient-centred strategies and detailed communication in pediatric dentistry for children with DS, providing valuable insights for managing similar challenges in dental care.

## 1. Introduction

Children's refusal of dental care can lead to the failure of dental treatment in pediatric dentistry practice [1]. A qualitative study conducted on kindergarten students in Bandung, Indonesia, in 2018, revealed that most children (78%) had not been taken to a dentist until kindergarten age, primarily because they refused when prompted by parents or exhibited resistant behaviour [2, 3]. The children's refusal of dental care is not solely due to dental fear but is more related to communication. Dentally anxious children do not all become uncooperative during treatment, especially when communication between the dentist, parents, and child flows smoothly [4, 5]. However, specific populations, such as children with special health care needs, will likely have challenges performing cooperative behaviour during dental treatment [6]. Down syndrome is one of the disorders that fall under special health care needs in children.

The dental management of children with DS generally is not different from that of other healthy children [7]. There is a commonality that good collaboration between dentists, parents, and patients is important [8]. However, limitations in visual and hearing abilities, as well as intellectual disabilities, can impact communication in dental health care [9, 10]. Some DS children may require more support from dental health care professionals than others [11]. This additional support can involve the approach taken by dental health professionals in assisting children with DS in receiving the dental care they deserve [7]. Therefore, this case report is aimed at presenting a nonpharmacological approach to supporting children with DS in receiving dental care.

## 2. Patient Characteristics

A 9-year-old boy named M arrived at the Dental and Oral Hospital of Universitas Padjadjaran (RSGM Unpad) accompanied by his mother, complaining of multiple cavities and retained primary teeth. The child has never visited a dentist before, and his mother expressed difficulty in cleaning M's teeth. A paediatrician diagnosed the child with Down syndrome at nine months, although the disability was already apparent since birth. Before the pandemic, the child had received speech therapy in the past and attended an inclusive elementary school. However, M attends a particular school for Down syndrome in the 4th grade. The child's intellectual condition has not been assessed.

The dental history revealed that the child's first tooth eruption occurred at six months, with an irregular sequence of eruptions compared to the expected age. M was bottle-fed until the age of two. He can eat the same food as other healthy children his age. M can brush his teeth but still requires assistance from his mother, who helps him brush twice daily in the morning and evening after bathing.

**Figure 1:**
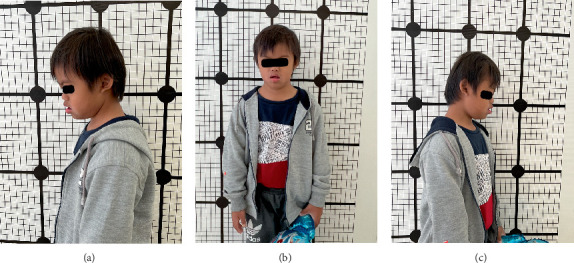
(a–c) Forward head posture of the patient.

The extraoral examination revealed no abnormalities in the temporomandibular joint and lymph nodes, hypertonicity of the lips, and a symmetrical face. M exhibited a forward head posture (Figure 1). Intraorally, the examination showed a normal labial frenulum and a standard number of teeth. There were no dental anomalies observed.

**Figure 2:**
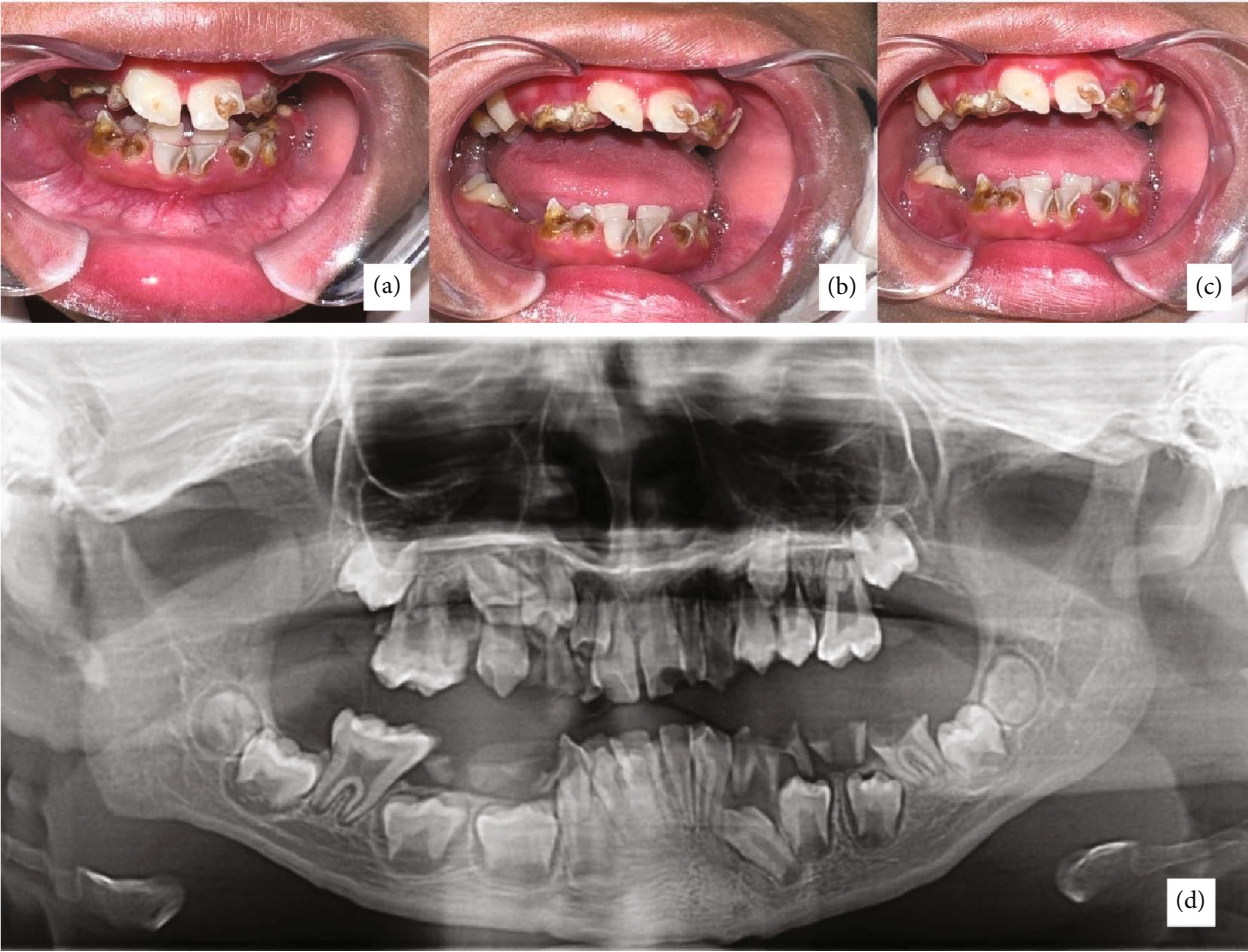
(a–c) Intraoral presentation and (d) panoramic feature.

The dental examination revealed the following conditions: gangrene of the root in 55, 53, 52, 64, 65, 36, 75, 74, 73, 83, 84, and 85; persistence of primary tooth 54, 63, 72, 71, 81, and 82; and reversible pulpitis in 16, 11, 21, 26, and 46. The intraoral condition of the teeth can be seen in Figure 2. The planned treatment includes extracting the affected teeth with a diagnosis of root gangrene (12 teeth) and the primary tooth persistence (6 teeth); composite filling for teeth 11 and 21; preventive resin restoration for teeth 16, 26, and 46; and topical application of fluoride. In addition, myofunctional therapy is also planned to optimize the oral motor function of the child.

## 3. Case Management

### 3.1. Dental Management

During the first visit, M refused and resisted entering the dental examination room. The operator initially interviewed the patient's mother to gather information. M would only sit in a chair near the exit and refused to sit in the dental chair. Additionally, M refused to be examined using dental instruments. However, M was willing to open their mouth and have extraoral and intraoral photos taken (Figure 2). M has limited speech abilities and tends not to speak much.

**Figure 3:**
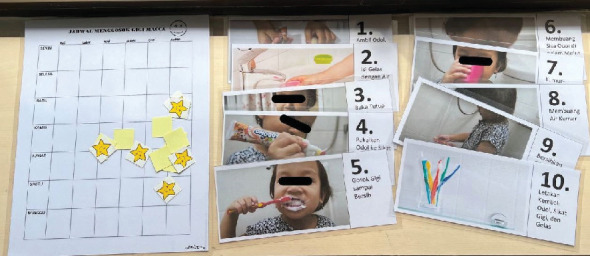
Toothbrushing sequence card.

During the second visit, which took place a week later, the dentist asked the parent to bring the child's toothbrush, toothpaste, and cup from home. The dentist prepared a sequence card demonstrating the steps for brushing teeth (Figure 3) and created a board where stickers could be placed as a reminder for brushing teeth in the morning and evening. When M entered, he sat in the same chair as the previous week. Further history taking revealed that M, when sitting in a car, would always choose the first seat he saw and refuse to move. Therefore, it was planned to remove all other chairs for future appointments, hoping M would sit in the dental chair. On this second visit, M was asked to look at pictures on a sequence card depicting the stages of brushing teeth. Then, the dentist guided M on how to brush his teeth based on the pictures shown one by one. M could follow the instructions and complete the brushing process in about 20 minutes. After the tell-show-do process of tooth brushing, M began to allow an intraoral examination using an oral mirror while standing, similar to the previous tooth brushing process. The sequence card was taken home and instructed to be used every time the patient brushed their teeth.

At the third visit, a scheduled dental visit flow is established to familiarise M, starting with tooth brushing and progressing to any necessary dental treatments. No chairs were available for M to sit on, so he remained standing. M's mother helped explain to him to sit in the dental chair, but he resisted and screamed. After several explanations and with the assistance of a doll demonstrating sitting in the dental chair, M finally agreed to sit there. The mother hugged M from behind and helped him get used to the semisupine position in the dental chair.

For the next visit planned for filling treatment, the dentist introduced the instruments that would be used, such as basic instruments, filling instruments, tri-way syringes, suction, and the dental unit light. The dentist expressed praise and gratitude every time M followed the given instructions. The dentist always involved M in every procedure as the leading performer, while the dentist acted more as an assistant to what M was doing.

Subsequent visits are planned with the same scenario sequence: removing all chairs from the examination room, including the dentist's stool chair. This is followed by tooth brushing, after which M is asked to sit in the dental chair. Once M is seated, the dentist's stool chair is brought into the room and occupied by the dentist. Dental treatments are tailored to the existing treatment plan. The tell-show-do approach consistently precedes all actions performed by the dentist.

The tooth preparation process for filling is initiated by introducing the bur instrument through an electric toothbrush. M is acquainted with the vibrations felt during the tooth preparation. Once M is comfortable with the electric toothbrush, the tooth drilling process is introduced through prophylactic brushing using a handpiece in the dental chair. The fifth visit completes the entire tooth-filling sequence.

Tooth extraction commenced during the sixth appointment, and six retained primary teeth were removed over three visits. The extraction procedure always started with introducing the instruments to be used. The Citoject® syringe was shown to M with the needle cap closed. The dentist introduced it as a “ballpoint pen” to mark the teeth to be extracted. With the cap still attached, the dentist demonstrated how the syringe worked and the clicking sound when the button was pressed to administer the anaesthetic. Dental forceps were introduced as a substitute for the dentist's fingers, as the dentist's fingers were too large to be used directly for tooth extraction. Aseptic measures, followed by topical anaesthetic gel, were performed before intraligamentary anaesthesia was administered to extract the mesial and distal sides of the teeth. After the extraction procedure, M was taught to bite on a gauze pad, and the parent was given postextraction instructions. On the next visit, there was a moment when M asked the dentist to administer local anaesthesia while video calling with his father. For the extraction of tooth 36, which required infiltration anaesthesia, the dentist first showed the aspirating syringe with the needle cap closed. This introduction was done after M underwent intraligamentary anaesthesia for several extraction procedures. The dentist referred to it as a “long ballpoint pen.”

**Figure 4:**
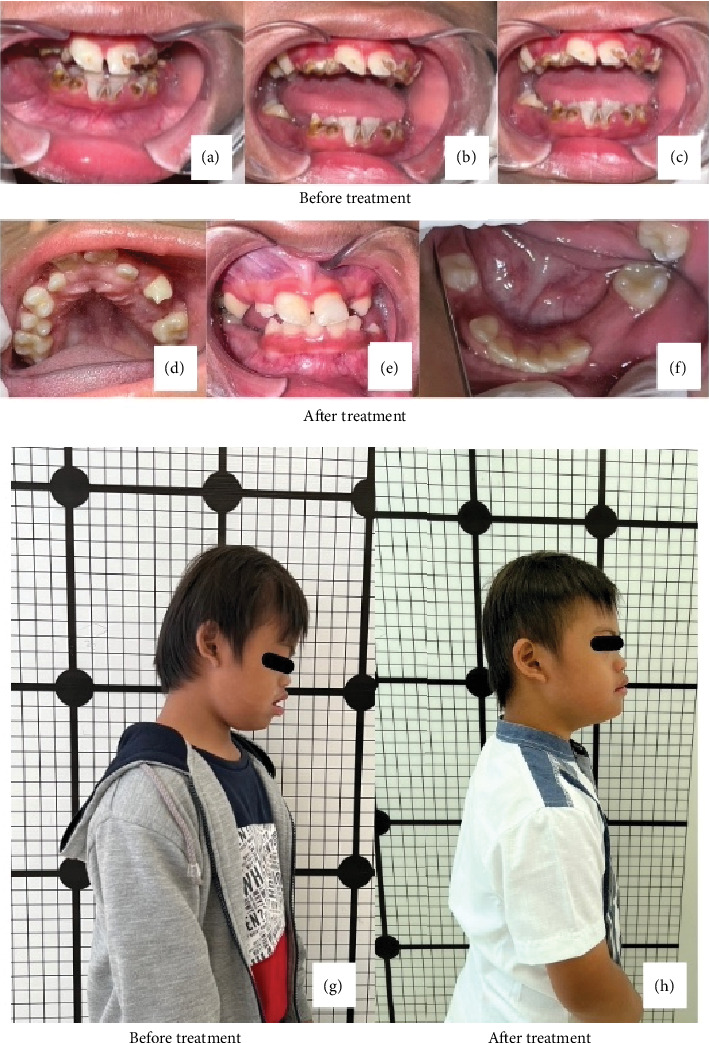
(a–c) Intraoral before treatment, (d, e) intraoral after treatment, and body posture presentation (g) before and (h) after treatment.

The entire dental treatment was carried out over 13 visits, spanning approximately 10 months—the final procedure on the teeth involved applying preventive resin restoration to the first three permanent molars (Figure 4). M was introduced to myofunctional therapy on the eighth visit to optimize oral muscle function. M always went home with a reward in the form of success stickers.

### 3.2. Oral Motoric Function Rehabilitation

**Figure 5:**
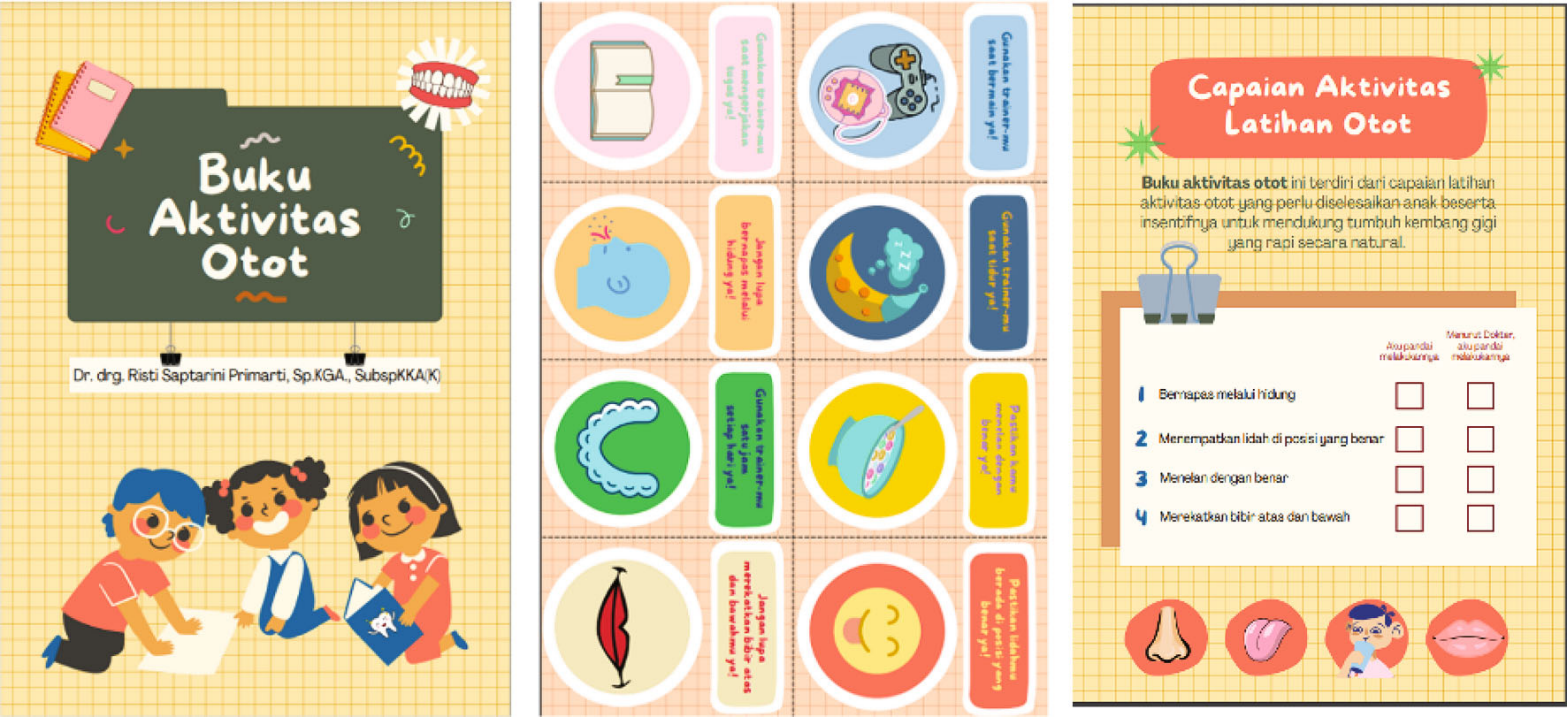
Oral Muscle Activity Handbook.

Myofunctional therapy was initiated concurrently with dental treatment from the ninth to the thirteenth visit. The dentist provided muscle therapy instructions and an *Oral Muscle Activity Handbook* (Figure 5) to the child's mother as the child's communication skills improved. The dentist taught the mother how to teach the child to close their lips tightly and breathe through their nose at home. This therapy was instructed to be done twice daily, each repeated ten times. The dentist also explained the proper tongue position to the child, with the tip resting behind the upper front teeth.

The myofunctional therapy began with breathing exercises and was followed by exercises for the lips, tongue, swallowing, chewing, and specific exercises for snoring if present. The sequence of breathing exercises that the child would perform is as follows:

1. The child needs to ensure breathing through the nose at a rate of 10-12 breaths per minute and swallowing with the correct and calm position
2. *Diaphragmatic Breathing.* Place one hand on the abdomen, stand in front of a mirror to observe the breathing movement using the abdomen, and control the movement of the chest. The exercise should be done for 2 minutes in each position: standing, sitting, and lying down. Repeat this Exercise 2-3 times a day
3. *Kids Breath Well Application (Aimed at Normalizing the Breathing Rate).* A child with hyperventilation can start the application at 15 breaths per minute for 2 minutes for two weeks. Gradually decrease the breaths per minute every two weeks until the child can comfortably breathe at ten breaths per minute. If it is challenging to reduce the rate, increase the duration first and then decrease the breaths per minute by 2-5 minutes. Repeat this exercise 2-5 times a day
4. *Nose Clearing.* The child sits with their feet on the floor and their lips closed. The child takes tiny breaths in and out. The child pinches their nose and continuously rocks their body from side to side until they feel the urge to breathe again. Release the nose pinch, and let the finger rest above the lip to help keep the lips closed. Then, breathe through the nose using the respiratory muscles. The child takes a short rest before starting the next exercise. Repeat this exercise 3-5 times in succession. The child should do this exercise every night before bedtime or when the nose is congested
5. *Paces.* The child is asked to pinch their nose, close their lips, and then take tiny breaths in and out. The child is instructed to start walking and count the steps they can take. When the child truly needs to breathe, they should stop, release the nose pinch, and breathe only through the nose. Place one hand on the abdomen to remind it to move as the child recovers. Wait until the child can breathe comfortably before continuing and then repeat the exercise for five rounds. Repeat this exercise twice a day. The speed should gradually increase every week, at least five times. The goal is to reach 60 to 80 steps. Once the goal is achieved, continue doing it once a week to maintain the achieved progress
6. *Pufferfish.* The child is asked to purse their lips, blow air into their cheeks, and then place their tongue at the “N” spot and hold it. While performing this exercise, the upper and lower teeth should not be touched. The child should breathe through the nose while holding the air in the cheeks. This exercise is done for 2-10 minutes and repeated thrice daily
7. *Tongue Carpet.* The child is asked to open their mouth wide and place the tongue at the base of the mouth. The child presses the tongue to the floor, opens the throat, and shows the uvula. The child can use a mirror to ensure the correct position. The child holds this position for 3 seconds, repeats it ten times per set, and performs it twice daily
8. *Beach Ball*. The child uses a beach ball placed on their stomach, with the ball against a wall. The child is asked to breathe through their nose. This exercise is done for 2 minutes and repeated twice. The child should ensure the movement is in the abdomen, not the chest while breathing

**Figure 6:**
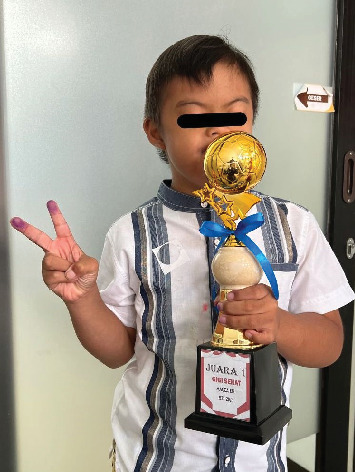
Championship trophy as reward for completion of treatments.

M's teeth were rechecked in the last visit, and topical fluoride was applied to all tooth surfaces. The oral condition appeared healthier, and there was an improvement in M's posture (Figure 4). The muscle therapy was continued at home, and a recall was planned once every month. As a reward for M's successful completion of the entire dental and oral muscle treatment, the dentist awarded M a trophy based on M's story during one visit, where M dreamed of receiving a championship trophy (Figure 6).

## 4. Discussion

Down syndrome (DS) is a genetic disorder caused by abnormalities in chromosome 21 and is the most common cause of intellectual disabilities [12]. Intellectual disabilities associated with DS lead to behavioural limitations, so oral health problems cannot be avoided, one of which is dental caries [7]. Individuals with special needs have a higher prevalence of oral diseases, mainly dental caries, and face challenges in maintaining oral hygiene. There is a higher risk of restorative failure, so dentists often opt for more aggressive treatment approaches. Dentists may also prefer to handle patients with less complicated procedures to prevent complications or treatment failure [13].

Patients with mental or physical disabilities often face difficulties accessing dental services and undergoing treatment. Patients who are uncooperative and have physical or mental impairments often require sedation or general anaesthesia for dental treatment. Sedation can be an effective and safe alternative to general anaesthesia for patients with disabilities. However, it may be challenging to administer sedation to uncooperative patients or those with respiratory obstructions or mouth breathers, which is typical in patients with DS. Dental treatments typically require patients to sit still and keep their mouths open for extended periods, presenting a challenge for dentists when caring for children with special needs. Another option for uncooperative children with special needs is general anaesthesia [13].

Indications for general anaesthesia in dental treatment include patients with medical disorders, those with difficulty cooperating, and those needing extensive dental care. The most common indication for general anaesthesia is a lack of cooperation and comorbidities. Other indications may include difficulties related to cooperation in patients with autism, dementia, intellectual disabilities, and other mental disabilities [13, 14].

During the initial examination, M showed resistance when asked to sit in the dental chair and keep their mouth open for a long time. M became angry and shouted when instruments were inserted into his mouth. M required extensive treatment for his oral problems, but M's uncooperative behaviour led to the consideration of performing the procedures under general anaesthesia. However, the patient's parents rejected this option due to cost concerns, and the parents were afraid of the potential risks associated with general anaesthesia, even though the safety factors of GA related to M's condition had been explained. The parents requested that the treatment be done conventionally in a dental chair with a nonpharmacological approach.

The dental office may encounter challenges when dealing with children whose parents exhibit a more permissive and less controlling approach to parenting. How parents raise their children directly influences the efficacy of behaviour management and the success of dental procedures [15]. The emotional environment for a child is shaped by parental attitudes, beliefs, and actions, which ultimately determine their parenting style. Different parenting styles result in varying behaviours from children, which play a crucial role in developing the child's ability to cope, potentially affecting their behaviour during dental visits and impacting the effectiveness of treatment [16–18].

From history taking, it is known that when M was diagnosed with DS, the patient's parents did not accept this, so they did not seek special medical treatment regarding the child's condition. The parents sought alternative treatment outside of health professionals if M experienced illness, including when he complained of toothache. Parents also said they tend to be permissive, agreeing to all their children's wishes to prevent M's tantrums. This parental behaviour can be seen when M refuses treatment; the mother will immediately pull the child close to her as if to provide protection.

The patient's treatment was conducted conventionally in the dental chair, spanning thirteen visits until mouth rehabilitation was completed. Several behaviour guidance techniques were employed: tell-show-do, desensitization, modelling, distraction, euphemisms, and behaviour shaping with positive reinforcement. Each visit served as preparation for the subsequent treatment. For example, in a prior visit before a filling procedure, the patient was introduced to the dental unit light, suction device, and tri-way syringe and familiarised with the vibrations of an electric toothbrush during tooth cleaning with a bur. These approaches align with several guidelines the American Academy of Pediatric Dentistry (AAPD) recommended [19].

The role of the mother is crucial in every aspect of treatment and muscle training. The patient is more cooperative when the mother explains, making it easier for the dental professional to perform the treatment. It is recommended that the first visit to the pediatric dentist be scheduled before the eruption of primary teeth so that parents and caregivers can be given appropriate instructions on oral hygiene and good eating habits to maintain children's oral health. Regular visits to the dentist are essential to monitor the growth of teeth and the craniofacial development of the child [20, 21].

## 5. Conclusion

This case report illustrates the successful management of dental care refusal in a child with Down syndrome, highlighting the complexities faced by pediatric patients with special health care needs. Comprehensive dental care was achieved through a strategic approach that emphasized behaviour guidance, patient communication, and the incremental introduction of dental procedures. The patient-centred approach, supported by effective collaboration among dental professionals, caregivers, and the patient's family, proved crucial in overcoming barriers and facilitating dental rehabilitation. This case reinforces the importance of adaptability and personalized care in pediatric dentistry, potentially guiding future strategies for similar patient populations.

## Data Availability

The data that support the findings of this report are available upon reasonable request. Researchers interested in accessing the data for replication or further analysis should contact Arlette Suzy Setiawan at arlette.puspa@unpad.ac.id. We are committed to promoting transparency and reproducibility in research and will make every effort to provide the data in a timely manner.

## Supplementary Materials

Supplementary Materials Supplementary Materials Parent's publication consent.
