# Supplementary material for: Navigating Treatment Refusal: Behaviour Guidance for Down Syndrome Oral Health Management
Source: Case Rep Dent. 2024 Jun 18;2024:2966972. doi: 10.1155/2024/2966972 (PMC11199061; doi:10.1155/2024/2966972)
Supplement: Supplementary Materials — Parent's publication consent. [file 2966972.f1.pdf]

Patient Consent Form

To record a patient's consent to publication of information relating to them or a relative, in a Wiley publication.

Name of patient: Macca Fayyaz Hazzazi

Title of publication/product: Navigating Treatment Refusal: Behavior Guidance for Down Syndrome Oral Health Management - A Case Study

Principal author/editor: Lila Sari

Principal author/editor's address:____

____

I, [Ahmad Zen Zaeni ~~NAME OF PATIENT /~~ PARENT / ~~GUARDIAN / RELATIVE~~***] (the "Licensor"), give my permission to use clinical information/video/photographic material relating to [Ahmad Zen Zaeni NAME AND RELATIONSHIP***] in the publication identified above to be published by John Wiley & Sons, Inc. or one of its affiliated companies ("Wiley"), such permission to extend to publication of the information by Wiley and its licensees in all media and languages throughout the world.

***In cases where the patient has died or is incapable of giving consent, consent may be given by the next of kin. If the patient is under the age of 16, consent should be given by a parent or guardian.

I understand that:

The information/video/photographic material will be used only in educational publications intended for health professionals

(1) My name will not be published and Wiley will endeavour to ensure that I cannot be identified from the clinical information, other than in relation to identifiable material (such as videos/photographic material) for which I give consent. However I also understand that there is a low possibility that I may be identified from the clinical information.

(2) If the publication or product is published on an open access basis, I understand that it may be accessed freely throughout the world.

This Agreement shall be governed by, and construed in accordance with: 1) the laws of England and Wales, if the Licensor is located outside of the United States, or 2) the laws of the State of New York, if the Licensor is located in the United States. In relation to any legal action or proceedings to enforce this Agreement or arising out of or in connection with this Agreement each of the parties irrevocably submits to the non-exclusive jurisdiction of the courts: 1) in England and Wales, if the Licensor is located outside of the United States, or 2) in New York, New York, if the Licensor is located in the United States.

*****SIGNATURE OF PATIENT/PARENT// GUARDIAN / NEXT OF KIN** ......[signature]......

*****IF PARENT / GUARDIAN / NEXT OF KIN, STATE RELATIONSHIP TO PATIENT** ......father......

[ADDRESS] Cisalatri Rt 03/06, no 115 kel Cipadung kec, Cibiru - Bandung 40614
____

____

____

[DATE] August 18, 2023

SIGNATURE OF HEALTH PROFESSIONAL OBTAINING PERMISSION (IF APPROPRIATE)

....

[ADDRESS] ____

____

____

[DATE]____

Note to principal author: The original signed consent form should be retained by the principal author.

Note to health professional: In addition to the consent form, please ensure that any other necessary permissions are cleared for use of the information, including any permissions required for use of information contained in medical records.
